# Evaluation of the INDICAID COVID-19 Rapid Antigen Test in Symptomatic Populations and Asymptomatic Community Testing

**DOI:** 10.1128/spectrum.00342-21

**Published:** 2021-08-04

**Authors:** Ricky Y. T. Chiu, Noah Kojima, Garrett L. Mosley, Kwok Kin Cheng, David Y. Pereira, Matthew Brobeck, Tsun Leung Chan, Jonpaul Sze-Tsing Zee, Harsha Kittur, Cheuk Yiu Tenny Chung, Eric Tsang, Kajal Maran, Raymond Wai-Hung Yung, Alex Chin-Pang Leung, Ryan Ho-Ping Siu, Jessica Pui-Ling Ng, Tsz Hei Choi, Mei Wai Fung, Wai Sing Chan, Ho Yin Lam, Koon Hung Lee, Sean Parkin, Felix C. Chao, Stephen Ka-Nung Ho, Daniel R. Marshak, Edmond Shiu-Kwan Ma, Jeffrey D. Klausner

**Affiliations:** a Phase Scientific International Ltd., Garden Grove, California, USA; b Phase Scientific International Ltd., Hong Kong, People’s Republic of China; c Department of Medicine, University of California Los Angeles, Los Angeles, California, USA; d Department of Pathology, Hong Kong Sanatorium and Hospital, Hong Kong, People’s Republic of China; e Department of Hospital Administration, Hong Kong Sanatorium and Hospital, Hong Kong, People’s Republic of China; f CityHealth Urgent Care, Alameda, California, USA; g Department of Preventive Medicine, University of Southern California Keck School of Medicine, Los Angeles, California; Children’s Hospital Los Angeles, University of Southern California

**Keywords:** COVID-19, SARS-CoV-2, rapid, antigen, asymptomatic screening, Hong Kong, USA

## Abstract

As the COVID-19 pandemic progresses, there is an increasing need for rapid, accessible assays for SARS-CoV-2 detection. We present a clinical evaluation and real-world implementation of the INDICAID COVID-19 rapid antigen test (INDICAID rapid test). A multisite clinical evaluation of the INDICAID rapid test using prospectively collected nasal (bilateral anterior) swab samples from symptomatic subjects was performed. The INDICAID rapid test demonstrated a positive percent agreement (PPA) and negative percent agreement (NPA) of 85.3% (95% confidence interval [95% CI], 75.6% to 91.6%) and 94.9% (95% CI, 91.6% to 96.9%), respectively, compared to laboratory-based reverse transcriptase PCR (RT-PCR) using nasal specimens. The INDICAID rapid test was then implemented at COVID-19 outbreak screening centers in Hong Kong as part of a testing algorithm (termed “dual-track”) to screen asymptomatic individuals for prioritization for confirmatory RT-PCR testing. In one approach, preliminary positive INDICAID rapid test results triggered expedited processing for laboratory-based RT-PCR, reducing the average time to confirmatory result from 10.85 h to 7.0 h. In a second approach, preliminary positive results triggered subsequent testing with an onsite rapid RT-PCR, reducing the average time to confirmatory result to 0.84 h. In 22,994 asymptomatic patients, the INDICAID rapid test demonstrated a PPA of 84.2% (95% CI, 69.6% to 92.6%) and an NPA of 99.9% (95% CI, 99.9% to 100%) compared to laboratory-based RT-PCR using combined nasal/oropharyngeal specimens. The INDICAID rapid test has excellent performance compared to laboratory-based RT-PCR testing and, when used in tandem with RT-PCR, reduces the time to confirmatory positive result.

**IMPORTANCE** Laboratory-based RT-PCR, the current gold standard for COVID-19 testing, can require a turnaround time of 24 to 48 h from sample collection to result. The delayed time to result limits the effectiveness of centralized RT-PCR testing to reduce transmission and stem potential outbreaks. To address this, we conducted a thorough evaluation of the INDICAID COVID-19 rapid antigen test, a 20-minute rapid antigen test, in both symptomatic and asymptomatic populations. The INDICAID rapid test demonstrated high sensitivity and specificity with RT-PCR as the comparator method. A dual-track testing algorithm was also evaluated utilizing the INDICAID rapid test to screen for preliminary positive patients, whose samples were then prioritized for RT-PCR testing. The dual-track method demonstrated significant improvements in expediting the reporting of positive RT-PCR test results compared to standard RT-PCR testing without prioritization, offering an improved strategy for community testing and controlling SARS-CoV-2 outbreaks.

## INTRODUCTION

The global SARS-CoV-2 pandemic has continued despite implementation of significant public health measures (1). Over 155 million worldwide cases of COVID-19 and over 3 million COVID-19 deaths have been reported as of May 2021. Rapid identification of SARS-CoV-2 infection, patient isolation, and contact tracing are essential for disease containment (2).

The current gold standard for detecting SARS-CoV-2 is reverse transcriptase PCR (RT-PCR) (3). While RT-PCR can detect nucleic acids from SARS-CoV-2 with high sensitivity, RT-PCR requires equipment and special training and can take days for results to be available following sample collection (4). Due to the transmissibility of SARS-CoV-2 (basic reproductive number *R*_0_ of 2.87), long turnaround times for results may lead to a high number of avoidable transmissions (5, 6).

In contrast, lateral flow immunoassays (LFAs) are an inexpensive testing solution that can be used at point-of-care settings, do not require laboratory equipment, and can generate results quickly. However, the performance of LFA-based SARS-CoV-2 rapid antigen tests in community testing settings can vary significantly (7–9).

In this study, we evaluated the clinical performance of the LFA-based INDICAID COVID-19 rapid antigen test (INDICAID rapid test) by PHASE Scientific International Ltd. A prospective multisite clinical study was performed in symptomatic patient populations in point-of-care (POC) community testing sites in the United States. The performance of the INDICAID rapid test was also evaluated in COVID-19 outbreak screening centers in Hong Kong as a part of an algorithm testing approach (termed “dual-track”) to screen for COVID-19-positive patients prior to RT-PCR testing in asymptomatic patient populations.

## RESULTS

### Prospective multisite clinical evaluation of the INDICAID COVID-19 rapid antigen test.

**San Francisco and Oakland population characteristics.** In total, 83 participants with at least two COVID-19 symptoms were enrolled at the San Francisco, CA and Oakland, CA sites. Two participants were excluded from the analysis due to lost samples during transport for RT-PCR. Of the 81 participant specimens analyzed, 44.4% were from female participants (Table S1), with a median age of 32 years (interquartile range [IQR], 25, 44). The most frequently reported symptoms were muscle/body ache (61.7%), congestion/runny nose (60.5%), fatigue (56.8%), and headache (53.1%). The breakdown of duration of symptoms was 1 to 2 days in 37 participants (45.7%), 3 to 4 days in 38 participants (46.9%), and 5 days in 6 participants (7.4%).

**San Fernando population characteristics.** In total, 270 participants with at least one COVID-19 symptom were enrolled at the San Fernando, CA site. Two participants were excluded from the analysis due to lost or spilled samples during transport for RT-PCR. Of the 268 participant specimens analyzed, 52.6% were from female participants (Table S2), and the median age was 35 years (IQR, 24, 50). The most frequently reported symptoms were sore throat (60.8%), headache (60.1%), congestion/runny nose (59.0%), and cough (54.9%). The distribution of duration of symptoms was 1 to 2 days in 109 participants (40.7%), 3 to 4 days in 127 participants (47.4%), and 5 days in 32 participants (11.9%).

**FIG 1:**
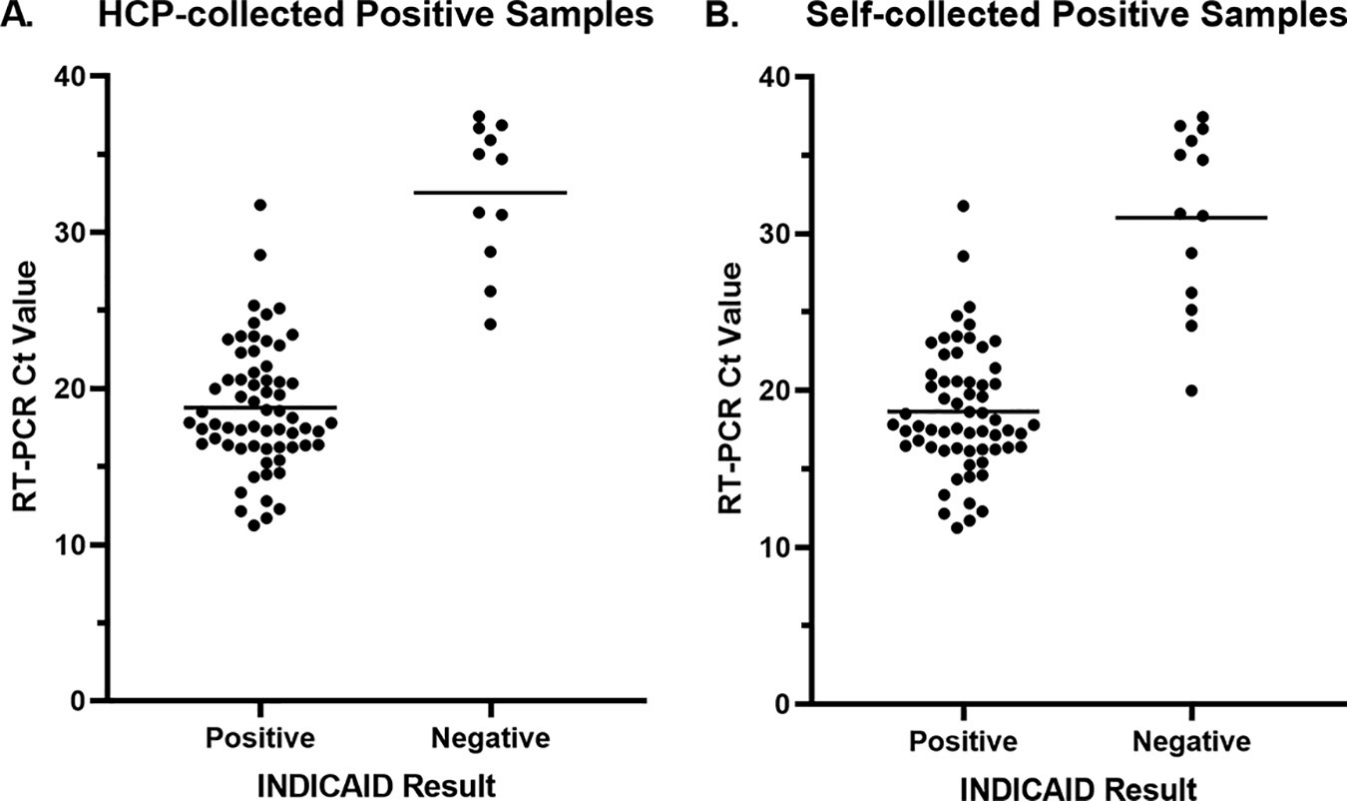
Association between *C_T_* value and INDICAID rapid test result when using (A) health care professional-collected samples and (B) self-collected samples for INDICAID rapid test.

**Performance of the INDICAID COVID-19 rapid antigen test in symptomatic patients.** Of the total 329 participant specimens included in the analyses, 75 tested positive with the comparator laboratory-based RT-PCR test. The mean cycle threshold value was 20.79 ± 6.39 (Fig. 1). The INDICAID rapid test demonstrated a PPA of 85.3% (95% confidence interval [95% CI], 75.6% to 91.6%) and an NPA of 94.9% (95% CI, 91.6% to 96.9%) when sample collection was conducted by a health care professional. There was a total of 11 false-negative INDICAID results that were not concordant with RT-PCR results; the mean threshold cycle (*C_T_*) value of the false-negative INDICAID specimens was 32.56 ± 4.59.

When sample collection was conducted by the participant (self-collected), the PPA was 82.7% (95% CI, 72.6% to 89.6%) and the NPA was 96.4% (95% CI, 93.4% to 98.0%). Overall percentage agreement (OPA) for health care provider (HCP)- and self-collected specimens was 92.8% and 93.4%, respectively (Tables 1 to 2,3,4).

**TABLE 1:** INDICAID COVID-19 rapid antigen test performance (HCP-collected sample) within 5 days of symptom onset against RT-PCR comparator method

No. INDICAID COVID-19 rapid antigen test samples:	No. comparative method (RT-PCR) samples:		
	Positive	Negative	Total
Positive	64	14	78
Negative	11	260	271
Total	75	274	349

**TABLE 2:** INDICAID COVID-19 rapid antigen test performance (HCP-collected sample) within 5 days of symptom onset against RT-PCR comparator method

Agreement between methods	Percent agreement	95% confidence interval	
		Lower limit	Upper limit
Positive percent agreement (PPA)	85.3%	75.6%	91.6%
Negative percent agreement (NPA)	94.9%	91.6%	96.9%
Overall percent agreement (OPA)	92.8%	89.6%	95.1%

**TABLE 3:** INDICAID COVID-19 rapid antigen test performance (self-collected sample) within 5 days of symptom onset against RT-PCR comparator method

No. INDICAID COVID-19 rapid antigen test samples	No. comparative method (RT-PCR) samples		
	Positive	Negative	Total
Positive	62	10	72
Negative	13	264	277
Total	75	274	349

**TABLE 4:** INDICAID COVID-19 rapid antigen test performance (self-collected sample) within 5 days of symptom onset against RT-PCR comparator method

Agreement between methods	Percent agreement	95% confidence interval	
		Lower limit	Upper limit
Positive percent agreement (PPA)	82.7%	72.6%	89.6%
Negative percent agreement (NPA)	96.4%	93.4%	98.0%
Overall percent agreement (OPA)	93.4%	90.3%	95.6%

### COVID-19 outbreak screening with the INDICAID COVID-19 rapid antigen test.

**FIG 2:**
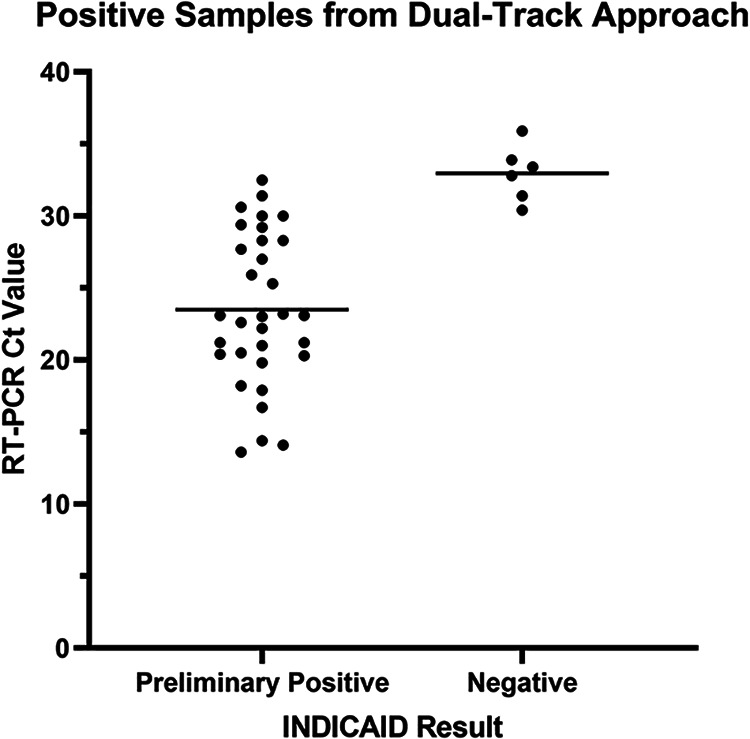
Association between *C_T_* value and INDICAID rapid test results when implemented in outbreak screening centers.

**Performance of the INDICAID COVID-19 rapid antigen test in asymptomatic patients.** In total, 22,994 asymptomatic individuals were screened at 12 outbreak screening centers in Hong Kong. Thirty-eight (38) of the total 22,994 patients tested positive for SARS-CoV-2 by laboratory-based RT-PCR. The INDICAID rapid test demonstrated a PPA of 84.2% and an NPA of 99.9% against the comparator RT-PCR method (Tables 5 and 6). All samples tested on site by the cobas SARS-CoV-2 & Influenza A/B nucleic acid test demonstrated concordant results with laboratory-based RT-PCR from ONCO Medical Laboratory. There were six false-negative INDICAID results that were not concordant with RT-PCR results; the mean *C_T_* value of the six samples was 32.97 ± 1.94 (Fig. 2).

**TABLE 5:** INDICAID COVID-19 rapid antigen test performance during implementation in outbreak screening centers in Hong Kong

No. INDICAID COVID-19 rapid antigen test samples	No. comparative method (RT-PCR) samples		
	Positive	Negative	Total
Positive	32	18	50
Negative	6	22,938	22,944
Total	38	22,956	22,994

**TABLE 6:** INDICAID COVID-19 rapid antigen test performance during implementation in outbreak screening centers in Hong Kong

Agreement between methods	Percent agreement	95% confidence interval	
		Lower limit	Upper limit
Positive Percent Agreement (PPA)	84.2%	69.6%	92.6%
Negative Percent Agreement (NPA)	99.9%	99.9%	100.0%
Overall Percent Agreement (OPA)	99.9%	99.8%	99.9%

**Time to positive result confirmation for each dual-track testing algorithm.** Time-to-result data from 8 of 10 positive samples in approach A and 12 of 18 positive samples in approach B were available for analyses. The time to result for the standard approach was estimated using 299 negative samples randomly selected across 3 days and multiple sites, as the extraction of reported time data from all 22,944 negative samples would be a labor-intensive process.

After a preliminary positive result with the INDICAID rapid test, both approach A and approach B demonstrated a time to positive confirmatory RT-PCR result shorter than that of the standard approach. Positive patient confirmation for approach A was on average 7.0 h (mean, 420 ± 151 min) compared to an average of 10.85 h (mean, 651 ± 171 min) for the standard approach [*t* statistic *t*(7) = 4.3, *P* < 0.004]. Similarly, for approach B, positive patient confirmation was on average 0.84 h (mean, 50.4 ± 14 min) compared to an average of 10.85 h (mean, 651 ± 171 min) for the standard approach [*t* statistic *t*(232) = 56.3, *P* < 0.0001].

## DISCUSSION

In the prospective clinical study in symptomatic patient populations, the INDICAID rapid test demonstrated a PPA of 85.3% and an NPA of 94.9% against comparator laboratory-based RT-PCR. False-negative results were observed mainly in participant specimens with higher *C_T_* values and likely lower viral loads. Given that participant-collected samples resulted in similar performance estimates as clinician-collected, preference for self-collection might be used in implementation to reduce risk and improve work flow at clinical or community testing sites.

When implemented for outbreak testing of asymptomatic patients, the INDICAID rapid test demonstrated a PPA and an NPA of 84.2% and 99.9%, respectively, against laboratory-based RT-PCR. The similarity of the PPA and NPA to those of the prospective clinical study suggest minimal or no loss in performance of the INDICAID rapid test when used in asymptomatic populations in a real-world setting. When combined with RT-PCR testing in the dual-track testing approach, the INDICAID rapid test was able to successfully reduce the time to confirmatory positive result from an average of 10.85 h to 0.84 h. The reduced time to positive patient identification and notification could result in a significant reduction in transmission in densely populated communities.

The performance of the INDICAID rapid test can be compared to that of other widely adopted rapid antigen tests for SARS-CoV-2 such as the BinaxNOW COVID-19 test by Abbott. The BinaxNOW test, which received emergency use authorization by the FDA, has manufacturer-reported PPA and NPA of 84.6% and 98.5%, respectively, compared to an RT-PCR comparator method. Independent studies performed by the CDC reported a PPA between 64.2 and 82% in symptomatic populations and a PPA between 35.8 and 52% in asymptomatic populations. NPA was greater than 98% in both populations (7, 10). A comparison of performance metrics suggests that the INDICAID rapid test may provide performance similar or superior to that of other widely adopted rapid antigen tests, particularly when utilized in asymptomatic populations. However, due to study differences in disease prevalence, comparator method, proportion of SARS-CoV-2 variants, and other factors, additional head-to-head studies will need to be performed.

There were limitations during the symptomatic clinical evaluation. The tests were conducted under controlled temperature and lighting conditions and interpreted by a limited pool of trained operators. Widespread community testing might occur under less-controlled conditions. Several point-of-care flex studies (see supplemental material) were performed to demonstrate that the INDICAID rapid test is robust under suboptimal conditions (i.e., extreme temperature and humidity, out-of-specification buffer addition, and variable result reading times).

Although the performance data suggest that the INDICAID rapid test has high sensitivity and specificity, some results that are discordant relative to those of the comparator PCR assays were still observed. Qualitative visual-based LFAs can present faint background lines that are subject to the interpretation of the test operator. In general, LFAs can be prone to false-positive signals due to higher background resulting from slower sample flow rates through the test device (11). Flow rates were not measured in this study but could vary from patient-to-patient sample.

Additionally, discordant results could be a result of sample collection order for each of the assays. Self-collected samples were always collected first, which might have affected the amount of virus/antigen remaining for the subsequent operator-collected samples. While the sample collection order is not expected to influence the comparator test result drastically, the effect of repeated sampling on the INDICAID rapid test has not been confirmed. Furthermore, since INDICAID rapid test samples and RT-PCR samples were sequentially collected and stored in separate collection tubes, variation in the amount of SARS-CoV-2 material found in individual samples may occur due to inconsistent sample collection. Differences in the quantity of target analyte present in each sample for the respective assays (viral antigens for INDICAID versus viral RNA for comparator PCR) may also be a contributing factor in the disparate results.

Due to certain limitations, no secondary testing was performed to elucidate the source of false-positive and false-negative test results. For example, at the U.S. clinical study sites, many of the samples did not have sufficient volume for repeat testing. Specimens eluted in the INDICAID test elution buffer also could not be analyzed by RT-PCR due to incompatibility of the buffer with the RT-PCR assay. During the outbreak testing in Hong Kong, the urgent nature of the testing setting did not provide an opportunity to further evaluate false-positive and false-negative samples.

Rapid antigen tests are expected to play an increasingly important role in COVID-19 testing programs. To our knowledge, our dual-track testing approach is one of the first widely coordinated efforts to integrate COVID-19 rapid antigen testing with rapid confirmatory RT-PCR testing in asymptomatic populations. However, testing with both rapid antigen and laboratory-based PCR for all asymptomatic individuals is not feasible from a resource perspective. Alternatively, screening with the INDICAID rapid test and reflexing to a rapid RT-PCR test for preliminary positive patients may be a viable strategy for asymptomatic population testing. We believe that our dual-track testing algorithm can be widely adopted in regions where the prevalence of COVID-19 is low and preventing new outbreaks is vital.

Recently, the United States CDC proposed similar screening algorithms that incorporate routine rapid antigen testing for asymptomatic populations, followed by preliminary positive patient confirmation by RT-PCR (12). The performance of the INDICAID COVID-19 rapid antigen test makes it a test suitable to be incorporated into such screening algorithms as communities, schools, and businesses reopen with the relaxation of public health measures. The dual-track testing approach may be an effective solution for COVID-19 surveillance and outbreak response, particularly in densely populated communities.

### Conclusion.

The INDICAID COVID-19 rapid antigen test demonstrated high PPA and NPA against laboratory-based RT-PCR in symptomatic and asymptomatic populations with various community rates of circulating SARS-CoV-2. Furthermore, when used in an algorithm testing approach (dual-track), the INDICAID COVID-19 rapid antigen test successfully reduced the time to confirmatory positive result. The dual-track testing approach highlights the advantages that rapid antigen testing can bring to already established RT-PCR testing frameworks. More studies are needed to determine optimal use of rapid antigen testing for screening algorithms.

## MATERIALS AND METHODS

### Description of the INDICAID COVID-19 rapid antigen test.

The INDICAID rapid test by PHASE Scientific is an LFA designed for the qualitative detection of SARS-CoV-2 nucleocapsid protein in nasal swab samples. The test produces a simple readout in 20 min with the presence of a visible test line to indicate detection of the SARS-CoV-2 antigen. The INDICAID rapid test achieves results without any additional equipment, power source, or special training.

**Sample collection and procedure of the INDICAID COVID-19 rapid antigen test.** To conduct an INDICAID rapid test, a nasal swab sample is collected by inserting the provided swab 1 in. into the nasal cavity. The swab is rubbed against the inside walls of both nostrils 5 times in a large circular path. The swab is then dipped into a buffer solution to elute the sample. Finally, 3 drops of the buffer solution-specimen mix are applied to the LFA test device. After 20 min, the user observes the test device for the presence or absence of a test line that indicates detection of the SARS-CoV-2 antigen. An internal quality control line is included to indicate whether the test has been performed correctly.

### Prospective multisite clinical evaluation of INDICAID COVID-19 rapid antigen test.

**Populations and study locations.** Between November 30, 2020 and January 8, 2021, study participants were enrolled at two U.S. clinical sites, CityHealth Urgent Care, San Francisco, CA and CityHealth Urgent Care, Oakland, CA. As part of the inclusion criteria, study participants were required to be at least 5 years of age and report onset of at least two of the following COVID-19 symptoms within 5 days or less: fever or chills, fatigue, sore throat, congestion or runny nose, cough, headache, diarrhea, shortness of breath or difficulty breathing, muscle or body aches, new loss of taste or smell, nausea, or vomiting. A patient enrichment strategy was implemented to increase the rate of SARS-CoV-2-positive participants enrolled at the CityHealth Urgent Care, Oakland, CA site. For this strategy, patients presenting at the clinic were first tested by CityHealth Urgent Care staff, as part of the standard of care, with an FDA emergency use approved (EUA) rapid antigen test (Quidel Sofia SARS antigen fluorescent immunoassay [FIA]) to prescreen potential subjects prior to study enrollment. CityHealth Urgent Care staff were asked to identify patients with preliminary positive or negative results for study screening. The trained study operators, blinded to the original patient standard-of-care result, performed specimen collection, processing, and testing. An equal number of positives and negatives were prescreened by the CityHealth Urgent Care staff at the CityHealth Urgent Care Oakland, CA site. Five unique study operators with various health care backgrounds (licensed medical assistants and registered nurses) conducted the study between the two sites.

Between February 6, 2021 and March 9, 2021, study participants were enrolled at a third U.S. clinical testing site (San Fernando Recreation Park in San Fernando, CA) who were at least 5 years of age and reported at least one of the COVID-19 symptoms listed above. Five unique study operators with various health care backgrounds (licensed medical assistants and registered nurses) conducted the study at the San Fernando, CA site. Results from any standard-of-care testing were not reported to the study investigators and were excluded from consideration or analysis.

**Testing procedure.** Patients were asked to provide a total of three nasal swab samples (not including samples collected for standard of care): a self-collected and observed nasal swab sample, followed by a second and a third nasal swab sample that were collected by the health care provider (HCP). For the first self-collected sample, the HCP provided specimen collection instructions and observed the specimen collection by the patient. The order of the second and third HCP-collected samples was randomized for testing with the investigational antigen test and the site’s RT-PCR comparator method to ensure that bias was not introduced due to unequal distribution of viral material. Immediately after sample collection, the samples for the RT-PCR comparator method were stored in viral transport media, while the other two nasal swabs were tested directly with the INDICAID rapid test according to the instructions for use (IFU).

INDICAID rapid test samples were tested immediately onsite after collection with no storage in accordance with the manufacturer’s protocol. Results of the INDICAID rapid test were interpreted by the test operators and recorded as positive, negative, or invalid based on the visual presence or absence of the control and test lines on the developed test strip. Test results were recorded after 20 min of assay development by one individual operator per participant.

At the Oakland, CA and San Francisco, CA study sites, swab samples collected for RT-PCR were stored in DNA/RNA Shield (Zymo Research, Irvine, CA), stored at room temperature, and shipped overnight at ambient temperature to Curative, Inc. (San Dimas, CA) per the manufacturer’s protocol for laboratory testing. The Curative SARS-CoV-2 assay, an FDA emergency use approved (EUA) test, was used as the RT-PCR comparator method (13).

At the San Fernando, CA study site, swab samples collected for RT-PCR were stored in BioCollections viral transport medium (VTM; BioCollections Worldwide, Inc., Miami, FL), stored on ice packs, and shipped overnight on ice packs to BioCollections Worldwide, Inc. per the manufacturer’s protocol for laboratory testing. BioCollections first performed the FDA EUA assay Hologic Aptima SARS-CoV-2 to determine the SARS-CoV-2 status of each patient sample. The remaining volume from positive specimens was then stored frozen at −70°C and later analyzed with the FDA EUA BioCollections worldwide SARS-CoV-2 assay to obtain cycle threshold (*C_T_*) results.

**Ethical approval.** To protect the rights, safety, and welfare of subjects, the study was conducted in accordance with 21 CFR 50, Protection of Human Subjects. Prior to study enrollment, each subject was asked to voluntarily provide his or her oral consent after being provided the institutional review board (IRB)-approved research information sheet. The onsite study investigator explained the nature, purpose, expected duration, and risks of study participation. Each potential subject had the opportunity to ask questions and receive answers from personnel conducting the study. All subjects were provided a copy of the research information sheet. The IRB was reviewed by Advarra, IRB number Pro00047510.

**Statistical analysis.** Difference testing for comparisons of groups in the population characteristics was performed with chi-square testing for categorical variables. Positive percentage agreement (PPA), negative percentage agreement (NPA), overall percentage agreement (OPA), and accuracy with 95% confidence intervals were calculated using Wilson-Score method. RT-PCR results were used as the comparator test. Analyses were performed on StataSE (StataCorp, College Station, TX). Figures were produced on GraphPad Prism 9.0.0 (GraphPad Holdings, La Jolla, CA).

### COVID-19 outbreak screening with the INDICAID COVID-19 rapid antigen test.

In this dual-track testing approach, the INDICAID rapid test was used to identify preliminary positives to trigger prioritization of sample processing for subsequent RT-PCR confirmatory testing. The outbreak testing was a collaborative effort between PHASE Scientific, ONCO Medical Laboratory, Hong Kong Sanatorium & Hospital, and the Food and Health Bureau of Hong Kong.

**Outbreak testing population and locations.** Twelve emergency outbreak testing centers (from December 10, 2020 to February 1, 2021) were organized at select locations in Hong Kong (Table 7). The sites were made available for asymptomatic individuals who perceived themselves as having a higher risk of exposure to SARS-CoV-2 or who were under compulsory testing requirements according to the guidelines from the Department of Health of the Government of the Hong Kong Special Administration Region.

**TABLE 7:** Dual-track testing locations in Hong Kong

Approach A locations	Approach B locations
Richland Gardens Apartments, Kowloon Bay	Maple Street Playground, Sham Shui Po
Tung Tau Estate, Chuk Un	Pei Ho Street Sports Center, Sham Shui Po
Jat Min Chuen Market, Shatin	Pitt Street location 1, Yau Ma Tei
Tsing Wah Playground, Tsing Yi	Pitt Street location 2, Yau Ma Tei
Tai Wo Hau Sports Center, Tai Wo Hau	
Li Chi Kok	
Kai Ching Estate, Kowloon City	
Princess Margaret Hospital, Kwai Chung	

**FIG 3:**
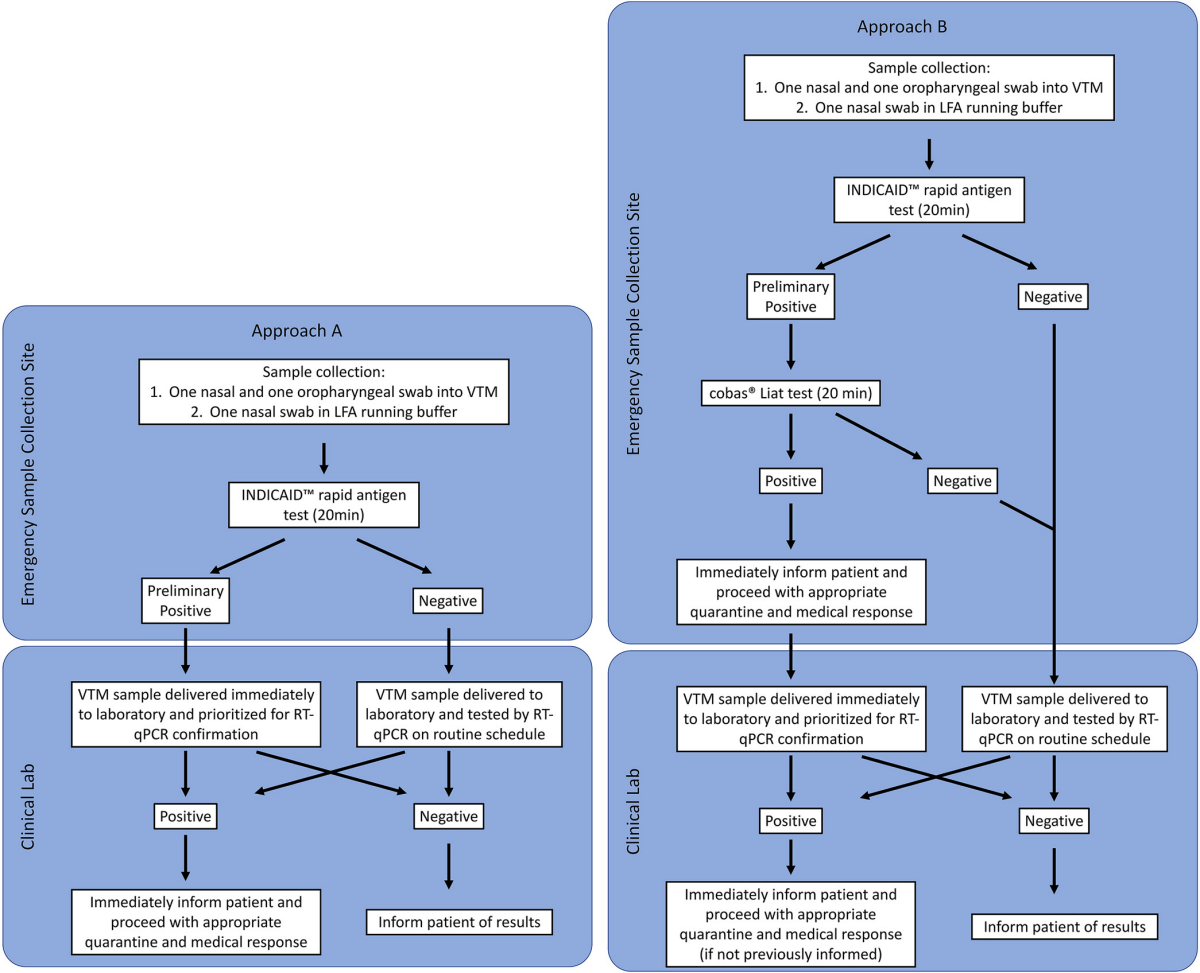
Flow charts of dual-track testing algorithm approaches A and B.

**Dual-track testing algorithms.** Two dual-track testing algorithms were implemented (Fig. 3). In both approaches, patient information was first collected at the registration station. Two nasal swab specimens and one oropharyngeal swab specimen were then collected by a clinician. One nasal swab specimen was used to perform the INDICAID rapid test immediately onsite. The additional nasal swab and oropharyngeal swab specimens were combined and stored in a single collection device containing viral transport medium (VTM) for subsequent RT-PCR testing in accordance with the Hong Kong Centre for Health Protection sample collection protocols.

In approach A, a preliminary positive result from the INDICAID rapid test would expedite the corresponding patient VTM sample for laboratory-based RT-PCR. Expedited samples resulted in the immediate transport of the individual sample to the ONCO Medical Laboratory compared to scheduled batched transportation for all other samples. Further, upon arrival to the laboratory, the expedited sample would be included in the next available round of RT-PCR testing. In approach B, a preliminary positive result from the INDICAID rapid test result would trigger the immediate testing of the corresponding patient VTM sample with an onsite rapid nucleic acid amplification test (cobas SARS-CoV-2 & Influenza A/B nucleic acid test on the cobas Liat system, Roche Molecular Diagnostics) to produce a confirmatory result. Results from the onsite rapid nucleic acid amplification test would typically be obtained within an additional hour. In addition to the onsite rapid RT-PCR test, the corresponding patient VTM sample would be transported to the ONCO Medical Laboratory for expedited laboratory-based RT-PCR testing in accordance with the requirements of the Hong Kong Centre for Health Protection. All samples testing negative with the INDICAID rapid test had their corresponding patient VTM samples processed according to the standard ONCO Medical Laboratory RT-PCR testing approach, in which samples were transported to the laboratory at regularly scheduled intervals and processed in the order of their arrival.

For both approaches, a positive RT-PCR test resulted in the immediate notification of the patient to isolate and take precautionary measures according to the guidelines of the Department of Health of the Government of the Hong Kong Special Administration Region. The RT-PCR result turnaround time for positive samples detected using approaches A and B were evaluated at 8 and 4 different emergency sample collection sites located in Hong Kong, respectively (Table 7).

**ONCO Medical Laboratory RT-PCR clinical laboratory testing procedure.** Total viral RNA was extracted from 200 μl of patient VTM sample using either prefilled 96-deep well plate (64T, Tianlong Technology) with automated GeneRotex 96 rotary nucleic acid extractor system (NANBEI, Tianlong Technology) or 96-well prepacked extraction reagents (SDK60104-96T, Bioperfectus Technologies) with automated nucleic acid extraction system (SSNP-3000A, Bioperfectus Technologies). RT-PCR was performed to determine the expression level of *orf1b* in the extracted RNA using PHASIFY DeCOVID SARS-CoV-2 RT-qPCR kit (3010100, Phase Scientific) according to the manufacturer’s protocol. Sample quality was validated via measuring expression levels of internal controls (viral, RNA-dependent RNA polymerase [*RdRP*]; human, *RNase P*). Positive and negative controls were included in each PCR.

**Statistical analysis.** Positive percentage agreement (PPA), negative percentage agreement (NPA), overall percentage agreement (OPA), and accuracy with 95% confidence intervals were calculated using Wilson-Score method. RT-PCR results were used as the comparator test. Analyses were performed on StataSE (StataCorp, College Station, TX). Figures were produced on GraphPad Prism 9.0.0 (GraphPad Holdings, La Jolla, CA).

For both dual-track testing approaches and the standard testing approach, mean and standard deviation were calculated for the elapsed time (minutes) from sample collection to confirmatory RT-PCR test result. A two-tailed, two-sample *t* test was performed comparing each of the dual-track approaches to the standard approach.
