# Supplementary material for: Evaluation of the INDICAID COVID-19 Rapid Antigen Test in Symptomatic Populations and Asymptomatic Community Testing
Source: Microbiol Spectr. 2021 Aug 4;9(1):10.1128/spectrum.00342-21. doi: 10.1128/spectrum.00342-21 (PMC8552729; doi:10.1128/spectrum.00342-21)
Supplement: SUPPLEMENTAL FILE 1 — Supplemental material. Download SPECTRUM00342-21_Supp_1_seq7.pdf, PDF file, 0.2 MB [file spectrum00342-21_supp_1_seq7.pdf]

Supplementary Information

Supplementary Table S1. Population characteristics from San Francisco, CA and Oakland, CA study sites. Percentage values (%) represent the percentage out of the total 81 participants in the cohort.

	Category	Total	(%)	SARS-CoV-2 Negative	(%)	SARS-CoV-2 Positive	(%)
n		81		44	54.3%	37	45.7%
Sex	Female	36	44.4%	22	27.2%	14	17.3%
	Male	45	55.6%	22	27.2%	23	28.4%
Age, years (median)		32		28		38	
Symptom Duration	1-2 days	37	45.7%	20	24.7%	17	21.0%
	3-4 days	38	46.9%	20	24.7%	18	22.2%
	5 days	6	7.4%	4	4.9%	2	2.5%
Symptoms	Fatigue	46	56.8%	24	29.6%	22	27.2%
	Fever or chills	33	40.7%	15	18.5%	18	22.2%
	Sore Throat	35	43.2%	21	25.9%	14	17.3%
	Nausea or vomiting	6	7.4%	6	7.4%	0	0.0%
	Congestion or runny nose	49	60.5%	30	37.0%	19	23.5%
	Cough	32	39.5%	14	17.3%	18	22.2%
	Headache	43	53.1%	26	32.1%	17	21.0%
	Shortness of breath or difficulty breathing	6	7.4%	5	6.2%	1	1.2%
	Diarrhea	13	16.0%	8	9.9%	5	6.2%
	Muscle or body aches	50	61.7%	26	32.1%	24	29.6%
	New loss of taste or smell	16	19.8%	8	9.9%	8	9.9%
Ct value (mean, s.d.)						20.00±5.80	
Ct value (median [IQR])						18.13 [16.29 – 22.96]	
Race	White	45	55.6%	26	32.1%	19	23.5%
	African American	11	13.6%	2	2.5%	9	11.1%
	Asian	12	14.8%	7	8.6%	5	6.2%
	Mixed	3	3.7%	2	2.5%	1	1.2%
	Native Hawaiian or Pacific Islander	1	1.2%	1	1.2%	0	0.0%
	Other	3	3.7%	2	2.5%	1	1.2%

	Not Provided	6	7.4%	4	4.9%	2	2.5%
Ethnicity	Hispanic, or Spanish origin	22	27.2%	9	11.1%	13	16.0%
	Not Hispanic, or Spanish origin	50	61.7%	27	33.3%	23	28.4%
	Not Specified	9	11.1%	8	9.9%	1	1.2%

Supplementary Table S2. Population characteristics from the San Fernando, CA study site. Percentage values (%) represent the percentage out of the total 268 participants in the cohort.

	**Category**	**Total**	**(%)**	**SARS-CoV-2 Negative**	**(%)**	**SARS-CoV-2 Positive**	**(%)**
n		268		230	85.8%	38	14.2%
Sex	F	141	52.6%	121	45.1%	20	7.5%
	M	127	47.4%	109	40.7%	18	6.7%
Age (median)		35					
Symptom Duration	1-2 days	109	40.7%	94	35.1%	15	5.6%
	3-4 days	127	47.4%	108	40.3%	19	7.1%
	5 days	32	11.9%	28	10.4%	4	1.5%
Symptoms	Fatigue	142	53.0%	126	47.0%	16	6.0%
	Fever or chills	100	37.3%	79	29.5%	21	7.8%
	Sore Throat	163	60.8%	140	52.2%	23	8.6%
	Nausea or vomiting	65	24.3%	58	21.6%	7	2.6%
	Congestion or runny nose	158	59.0%	136	50.7%	22	8.2%
	Cough	147	54.9%	117	43.7%	30	11.2%
	Headache	161	60.1%	136	50.7%	25	9.3%
	Shortness of breath or difficulty breathing	61	22.8%	54	20.1%	7	2.6%
	Diarrhea	30	11.2%	29	10.8%	1	0.4%
	Muscle or body aches	128	47.8%	110	41.0%	18	6.7%
	New loss of taste or smell	42	15.7%	31	11.6%	11	4.1%
Ct value (mean, s.d.)						21.57±6.86	
Ct value (median [IQR])						19.55 [17.07 – 24.35]	
Ethnicity	White	18	6.7%	17	6.3%	1	0.4%
	African American	10	3.7%	8	3.0%	2	0.7%

Asian	9	3.4%	9	3.4%	0	0.0%
Mixed	1	0.4%	1	0.4%	0	0.0%
Hispanic or Latino	225	84.0%	191	71.3%	34	12.7%
Not Provided	5	1.9%	4	1.5%	1	0.4%

Supplementary Table S3. Manufacturer-reported analytical limits of detection (LoD) for each comparator RT-PCR assay used in the Oakland, San Francisco, and San Fernando clinical study sites.

**Comparator Assay**	**Analytical LoD (cp/mL)[1]**	**LoD with FDA SARS-CoV-2 Reference Panel (NDU/mL)[2]**
Curative SARS-CoV-2	200	18000
Hologic Aptima SARS-CoV-2	83	600
Biocollections Worldwide SARS-CoV-2	1	1800

[1] In Vitro Diagnostics EUAs - Molecular Diagnostic Tests for SARS-CoV-2. https://www.fda.gov/medical-devices/coronavirus-disease-2019-covid-19-emergency-use-authorizations-medical-devices/in-vitro-diagnostics-euas-molecular-diagnostic-tests-sars-cov-2#imft2

[2] SARS-CoV-2 Reference Panel Comparative Data. https://www.fda.gov/medical-devices/coronavirus-covid-19-and-medical-devices/sars-cov-2-reference-panel-comparative-data#table2a

Analytical Validation of the INDICAID™ COVID-19 Rapid Antigen Test

Materials and Methods

Analytical limit of detection

The limit of detection (LoD) was determined by limiting dilution studies using characterized gamma-irradiated SARS-CoV-2 virus (BEI Resources, NIAID, NIH, SARS-Related Coronavirus 2, Isolate USA-WA1/2020, Gamma-Irradiated, NR-52287) spiked into pooled human nasal matrix from healthy donors (IRHUNF1ML, Innovative Research, MI, USA). At each dilution, 50 µL of sample was inoculated onto swabs and then assayed using the INDICAID™ COVID-19 Rapid Antigen Test procedure. An initial range finding study was performed using a 10-fold dilution series of the characterized SARS-CoV-2, testing the device in triplicate at each concentration. Concentrations between the last dilution that produced three positive test results and the first dilution to produce at least one negative test result were further evaluated using a 2-fold dilution series, in triplicate for each level, to refine the tentative LoD.

This LoD was then confirmed by testing 20 replicates with concentrations at the refined tentative limit of detection. The final LoD of the test was determined to be the lowest concentration resulting in positive detection of at least 19 out of 20 replicates.

To correlate the performance of the INDICAID™ Rapid Test with cycle threshold value output, 14 concentrations of SARS-CoV-2 from heat inactivated SARS-Related Coronavirus 2 Culture Fluid (1.02x 10^8 $TDID_{50}$/mL, 0810587CFHI, lot 325309, Zeptometrix) were prepared through serial dilution in the INDICAID™ Rapid Test buffer and then tested with both the INDICAID™ Rapid Test and with *ONCO Medical Laboratory RT-PCR method*. A dilution scheme that simulated the differences in dilution ratios between the INDICAID™ Rapid Test and the ONCO Medical Laboratory RT-PCR test was used. Viral-free test buffer was included as negative controls. The INDICAID™ Rapid Test was performed using 75µl of sample immediately after the dilution. Positive and negative band determinations were made by visual inspection from three blinded observers according to a standardized line intensity reference chart. Tests were analyzed at 20 minutes.

To determine the RT-PCR cycle threshold vales of the contrived samples, total viral RNA was extracted from 200µl of sample using 96-well pre-packed extraction reagents (SDK60104-96T, Bioperfectus Technologies) with automated nucleic acid extraction system (SSNP-3000A, Bioperfectus Technologies). The expression level of *Orf1b* in the extracted RNA was determined using PHASIFY™ DeCOVID SARS-CoV-2 RT-qPCR Kit (3010100, Phase Scientific) according to the manufacturer's protocol. Sample quality was validated via measuring expression levels of internal controls (viral: *RdRP*; human: *RNase P*). Positive and negative controls were

included in each PCR reaction. Non-linear regression analysis was performed on GraphPad Prism 9.0.0 fit to a sigmoidal curve constraining the top plateau at a rapid antigen test line intensity 12.

Endogenous interference, cross-reactivity, microbial interference

After the LoD was determined, evaluations of endogenous interference, cross-reactivity, and microbial interference were conducted according to the US FDA's Emergency Use Authorization (EUA) template for SARS-CoV-2 antigen test manufacturers. (1)

Flex studies for out-of-specifications test performance

A thorough hazard analysis was conducted to evaluate the impact of errors, or out-of-specifications conditions, on the rapid antigen test performance. To test the effect of extreme environmental conditions, contrived samples of 5.6×10^3 $TCID_{50}$/mL (2x the determined analytical LoD) gamma-irradiated SARS-CoV-2 in pooled nasal matrix, as well as non-spiked negative pooled nasal matrix, were tested on the INDICAID™ Rapid Test in low temperature (2-8°C) and high temperature/high humidity (40°C and near 95% relative humidity (RH)) conditions. One hour prior to the study, test kits were placed in a refrigerator maintaining 2-8°C or an incubator maintaining 40°C and 95% RH. Contrived samples were then applied to the test devices and allowed to run for 20 min in the same respective environments (n=3 per condition). Test results were recorded after 20 min.

To test the effect of INDICAID™ Rapid Test buffer volume variability, contrived samples of 5.6×10^3 $TCID_{50}$/mL gamma-irradiated SARS-CoV-2 in pooled nasal

matrix, as well as non-spiked negative pooled nasal matrix, were tested on the INDICAID™ Rapid Test at room temperature. Following the release of contrived specimen from the inoculated swab into the buffer solution, solution volumes of 1, 2, 3, 4, 5, 6 drops, and the entire buffer volume were applied to the test device (n=3 per condition). Test results for all replicates were interpreted at 20 minutes.

To test the effect of variable result read times, contrived samples of 5.6 x 10^3 $TCID_{50}$/mL gamma-irradiated SARS-CoV-2 in pooled nasal matrix, as well as non-spiked negative pooled nasal matrix, were tested on the INDICAID™ Rapid Test at room temperature. Test results were interpreted at 5, 10, 15, 20, 30, and 60 minutes after samples had been applied to the test device (n=3 per condition).

RESULTS

Analytical limit of detection

For the initial LoD range finding study, 10-fold serial dilutions of gamma-irradiated SARS-CoV-2 in pooled human nasal matrix were prepared with the highest test concentration of 2.8 x10^5 $TCID_{50}$/mL (1.4 x 10^4 $TCID_{50}$/swab). From this dilution series, the lowest concentration to produce 3 out of 3 positive results on the INDICAID™ Rapid Test was 2.8 x10^3 $TCID_{50}$/mL (1.4 x 10^2 $TCID_{50}$/swab). This tentative LoD was further refined using 2-fold serial dilutions between 2.8 x10^3 $TCID_{50}$/mL (1.4 x 10^2 $TCID_{50}$/swab) and 1.75 x 10^2 $TCID_{50}$/mL (8.75 $TCID_{50}$/swab). From this 2-fold dilution series, a concentration of 2.8 x 10^3 $TCID_{50}$/mL (1.4 x 10^2 $TCID_{50}$/swab) continued to be the lowest concentration that produced 3 out of 3 positive results. This concentration was confirmed to be the final LoD as 20 out of 20

replicates produced a positive result with test samples containing 1.4 x 10^2 $TCID_{50}$/swab.

The LoD in relation to cycle threshold value of the INDICAID™ Rapid Test was evaluated using contrived samples of varying concentrations of heat inactivated SARS-CoV-2 spiked into the INDICAID™ Rapid Test buffer. Results were reported in line intensity values from a standardized line intensity reference chart by three trained readers in a blind experimental design. Line intensity values correspond to the visibility of the test line to the user with 0 representing no visible test line and 12 representing the maximum test line intensity (Figure S1). Based on an industry standard for visually based lateral-flow immunoassays, a line intensity of 3 was utilized as the visual cut-off for the intended user (non-laboratory healthcare professionals). The visual cut-off intersects the non-linear regression line (R^2 = 0.976) at a Ct value of 27.2.

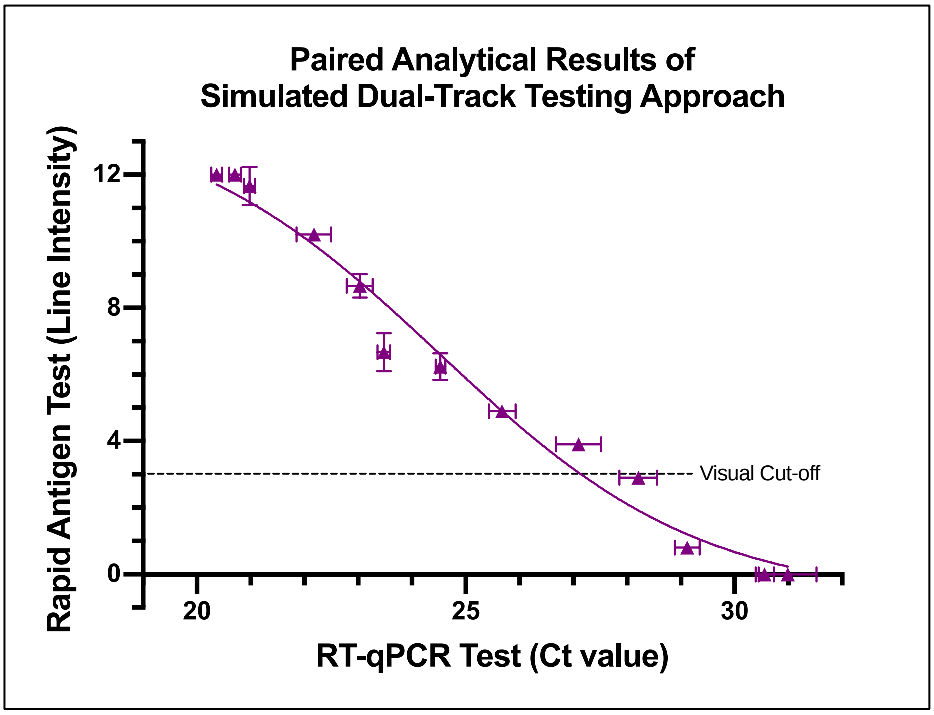

Supplementary Figure S1. Correlation of INDICAID™ COVID-19 Rapid Antigen Test and RT-PCR Ct results. Non-linear regression analysis was performed (R^2 = 0.976). All error bars are standard deviation.

Endogenous interference, cross-reactivity, microbial interference

No cross-reactivity nor test interference were observed for 27 common respiratory pathogens and pooled nasal wash in the presence of gamma-irradiated SARS-CoV-2 at 3x the analytical LoD. Furthermore, 14 endogenous substances that may be found in respiratory specimens of patients symptomatic for respiratory illness demonstrated no significant test interference in the presence of gamma-irradiated SARS-CoV-2 at 3x the analytical LoD.

Flex studies for out-of-specifications test performance

A series of flex studies was conducted to evaluate the influence of errors that can occur in point-of-care environments. These errors include extreme temperatures, extreme humidity, higher- and lower-than-recommended buffer volumes added to the test device, and sooner- and later-than-recommended read times. When tested with contrived samples in low temperature conditions (2-8°C) as well as high temperature and humidity conditions (40°C and 95% RH), the INDICAID™ Rapid Test produced expected positive and negative results and no invalid test results were observed. With low positive contrived samples (i.e., 2x the analytical LoD), accurate test result interpretation could be made by trained users as soon as 10 min and as late as 60 min after samples have been applied to the test device. Furthermore, accurate results were produced when 2-6 drops of the INDICAID™ Rapid Test buffer are applied to the test device, as opposed to the manufacturer-recommended 3 drops.

REFERENCES

1. FDA. 2020. Policy for Coronavirus Disease-2019 Tests During the Public Health Emergency (Revised): Immediately in Effect Guidance for Clinical Laboratories, Commercial Manufacturers, and Food and Drug Administration Staff.
